# Automated Analysis and Reannotation of Subcellular Locations in Confocal Images from the Human Protein Atlas

**DOI:** 10.1371/journal.pone.0050514

**Published:** 2012-11-30

**Authors:** Jieyue Li, Justin Y. Newberg, Mathias Uhlén, Emma Lundberg, Robert F. Murphy

**Affiliations:** 1 Center for Bioimage Informatics and Department of Biomedical Engineering, Carnegie Mellon University, Pittsburgh, Pennsylvania, United States of America; 2 Department of Biotechnology, AlbaNova University Center, Royal Institute of Technology, Stockholm, Sweden; 3 Science for Life Laboratory, Royal Institute of Technology, Solna, Sweden; 4 Lane Center for Computational Biology and Departments of Machine Learning and Biological Sciences, Carnegie Mellon University, Pittsburgh, Pennsylvania, United States of America; 5 Faculty of Biology and Freiburg Institute for Advanced Studies, Albert Ludwig University of Freiburg, Freiburg, Germany; Macquarie University, Australia

## Abstract

The Human Protein Atlas contains immunofluorescence images showing subcellular locations for thousands of proteins. These are currently annotated by visual inspection. In this paper, we describe automated approaches to analyze the images and their use to improve annotation. We began by training classifiers to recognize the annotated patterns. By ranking proteins according to the confidence of the classifier, we generated a list of proteins that were strong candidates for reexamination. In parallel, we applied hierarchical clustering to group proteins and identified proteins whose annotations were inconsistent with the remainder of the proteins in their cluster. These proteins were reexamined by the original annotators, and a significant fraction had their annotations changed. The results demonstrate that automated approaches can provide an important complement to visual annotation.

## Introduction

Knowledge of the subcellular locations of proteins provides critical context necessary for understanding their functions within the cell. Hence the field of location proteomics is concerned with capturing informative and defining characteristics of subcellular patterns on a proteome-wide basis [1], [2]. Automated methods for systematic study of protein locations, which combine fluorescence microscopy techniques with pattern recognition and machine learning algorithms, have been extensively described [1], [3]–[6]. Most of these studies involve extracting subcellular location features (SLFs) from images or cells [5], [7]. Automated analysis of subcellular patterns has been described for a proteome-scale image collection for yeast [8] and for a wide range of human tissues [9].

The latter study used images generated for thousands of proteins by the Human Protein Atlas (HPA, http://proteinatlas.org) using immunohistochemistry methods [10]. More recently, the HPA has been expanded to include images of cultured cells obtained by confocal immunofluorescence microscopy [11], [12]. We have previously described preliminary results demonstrating the feasibility of performing automated analysis of these confocal images [5]. These images have been annotated by visual inspection with specific terms describing subcellular location patterns.

**Figure 1:**
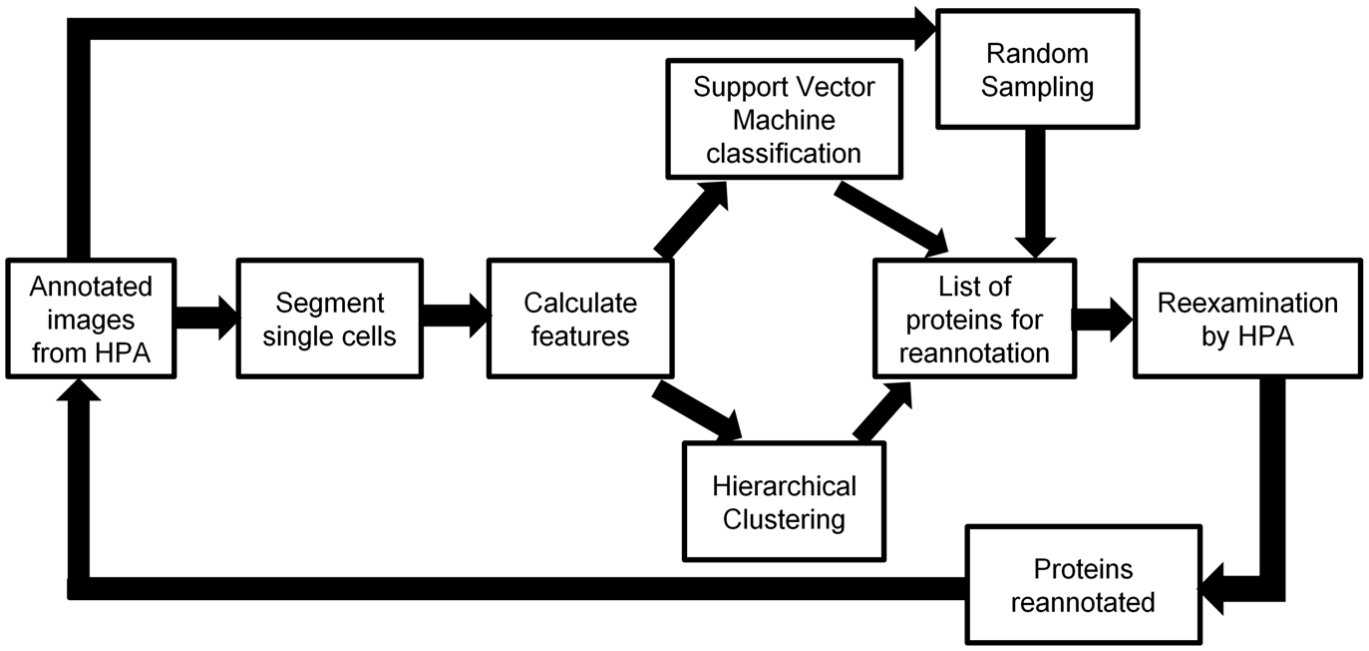
An overview of the framework introduced in this paper. We first collect immunofluorescence images from confocal microscopy that are annotated visually by HPA. These image fields are then segmented into single cells and various features (SLFs) are calculated. We then do two parallel analyses on the features. One is a supervised classification using a Support Vector Machine, and the other is an unsupervised hierarchical clustering. This results in two lists of proteins which have high probabilities of needing reannotation. Combined with another list of proteins sampled randomly, a final list which contains only the protein IDs are reexamined by the HPA annotation group. The annotations of some proteins may change, and these modified annotations can be incorporated in another cycle of analysis.

In this paper, we have extended this approach to include more classes and more proteins using a supervised learning approach, and added unsupervised learning to complement it. Based on these approaches, we furthermore identified proteins whose annotations appeared at odds with those of similar proteins. Re-examination of the images of these proteins revealed that a considerable number had been incorrectly annotated. Thus, our approaches can be used not only for the purpose of annotations de novo, but also for improving the accuracy of human annotations. An overview of the whole framework is shown in **Figure 1**.

## Results

In the following sub-sections, we present our results for two rounds of analyses. The first round and second round are consecutive with the same framework of analysis shown in **Figure 1**. They only differ in that they deal with two different but successive releases (4.0 and 5.0 respectively) of datasets from HPA.

### Automated Selection of Proteins for Reannotation

We began by segmenting confocal immunofluorescence images from the A-431 cell line in release 4.0 of HPA. These images had been previously annotated as being present in one or more subcellular locations by visual examination. The dataset contained images for 1551 proteins, of which 878 were localized specifically (solely) to one of eleven major subcellular location patterns (classes): centrosome, cytoplasm, cytoskeleton, endoplasmic reticulum, Golgi, lysosome/peroxisome/endosome, mitochondria, nucleoli, nucleus, nucleus without nucleoli, and plasma membrane. The number of proteins per class ranged from five to 326. We termed these single pattern proteins, and others which localized to more than one organelle as mixed pattern proteins. The ability of a Support Vector Machine (SVM) to recognize the eleven classes was estimated by nested five-fold cross-validation using the single pattern proteins.

The confusion matrix is shown in **Table 1**, with an overall accuracy of 82.4%. Despite the use of class-based weighting during training, it is clear that classes with fewer proteins have lower accuracies. It is also clear that plasma membrane and cytoplasmic patterns are difficult to distinguish using our current feature set (and that some cytoskeletal proteins are also misclassified as cytoplasmic).

**Table 1:** Classification results before first round of reannotation.

	centro.	cyto.	cytosk.	er	golgi	l/p/e	mitoch.	nucleoli	nucleus	w/o	PM
Centrosome (12)	**0.42**	0.08	0	0	0.33	0	0.08	0	0	0.08	0
Cytoplasm (326)	0	**0.97**	0.01	0	0	0	0.02	0	0	0.01	0
Cytoskeleton (37)	0	0.51	**0.46**	0	0	0	0.03	0	0	0	0
ER (34)	0	0.18	0	**0.76**	0	0	0.06	0	0	0	0
Golgi (41)	0	0.02	0	0	**0.9**	0	0.05	0	0	0.02	0
lys/pero/endo (26)	0	0.15	0	0	0.04	**0.62**	0.15	0	0.04	0	0
Mitochondria (104)	0.01	0.13	0	0	0.01	0	**0.86**	0	0	0	0
Nucleoli (37)	0	0	0	0	0	0	0	**0.92**	0	0.08	0
Nucleus (87)	0	0	0	0	0	0.02	0	0.06	**0.34**	0.57	0
Nucleus w/o nucleoli (167)	0	0	0	0	0	0	0	0.01	0.08	**0.92**	0
Plasma membrane (7)	0	0.86	0	0	0	0	0.14	0	0	0	**0**

Cell level feature classification confusion matrix. Bold values indicate agreement between the classifier and the true class. Overall classification accuracy is 82.4%. The number of proteins in each class is shown in parenthesis after the class name.

Using this classification approach, we can generate a list of proteins whose assignment by the classifier does not match the human annotation. There are many potential reasons for a protein being misclassified. A protein’s pattern may be different from those of most of the others in its class (e.g., a protein found only in the rims of Golgi cisternae may be annotated as Golgi along with many other proteins yet have a distinctly different pattern from the perspective of image analysis). Misclassfication may also occur if the features used to request the patterns do not capture subtle differences. Of course, some misclassification may result from incorrect annotation of the images. We therefore sought to identify proteins that we estimated as having a high probability of being incorrectly annotated. Using the approach described in Materials and Methods, we generated a list of 99 proteins for reannotation.

Since this supervised learning procedure relies on the human annotations to define classes, we also sought to use an unsupervised approach to group proteins by their patterns. Thus we implemented an alternative approach to identifying reannotation candidates by hierarchical clustering of single pattern proteins (see Materials and Methods). The optimal number of clusters determined by the Akaike information criterion was 56, and when proteins were assigned the dominant annotation of their cluster, an accuracy of 67% was obtained. We considered proteins that were included in a cluster containing mostly proteins from other classes as good candidates for reannotation. Using the fairly tight criteria described in the Methods, only 12 proteins were identified for reannotation by this approach.

### Reannotation and Retraining

**Figure 2:**
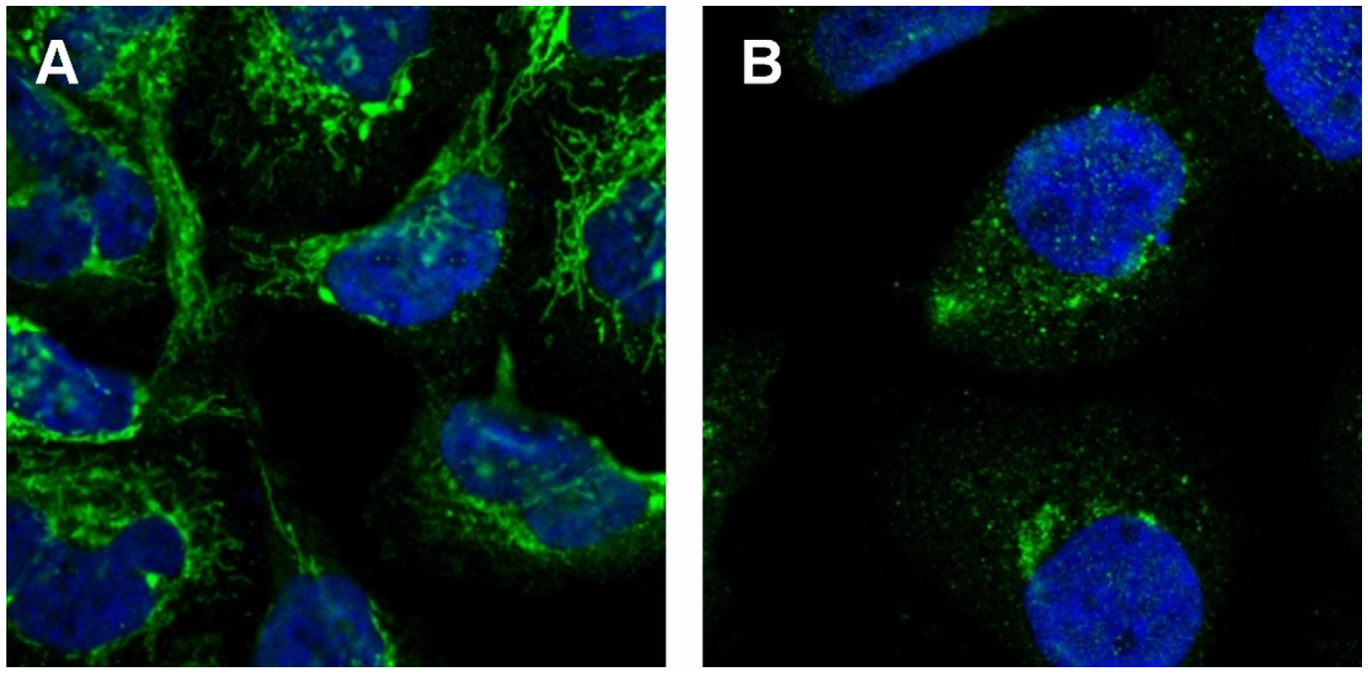
Examples of mis-annotated proteins identified by the SVM classification reannotation algorithm. (a) Protein “Thiosulfate sulfurtransferase” was identified in the first round analysis. The protein was visually annotated as “cytoskeleton” but was classified as “mitochondria” by an SVM classifier. The latter annotation was found to be correct upon re-examination. (b) Protein “proline-rich transmembrane protein 2″ was identified in the second round analysis. The protein was visually annotated as “cytoplasm” but was classified as “Golgi” by an SVM classifier. The latter annotation was found to be correct upon re-examination.

**Figure 3:**
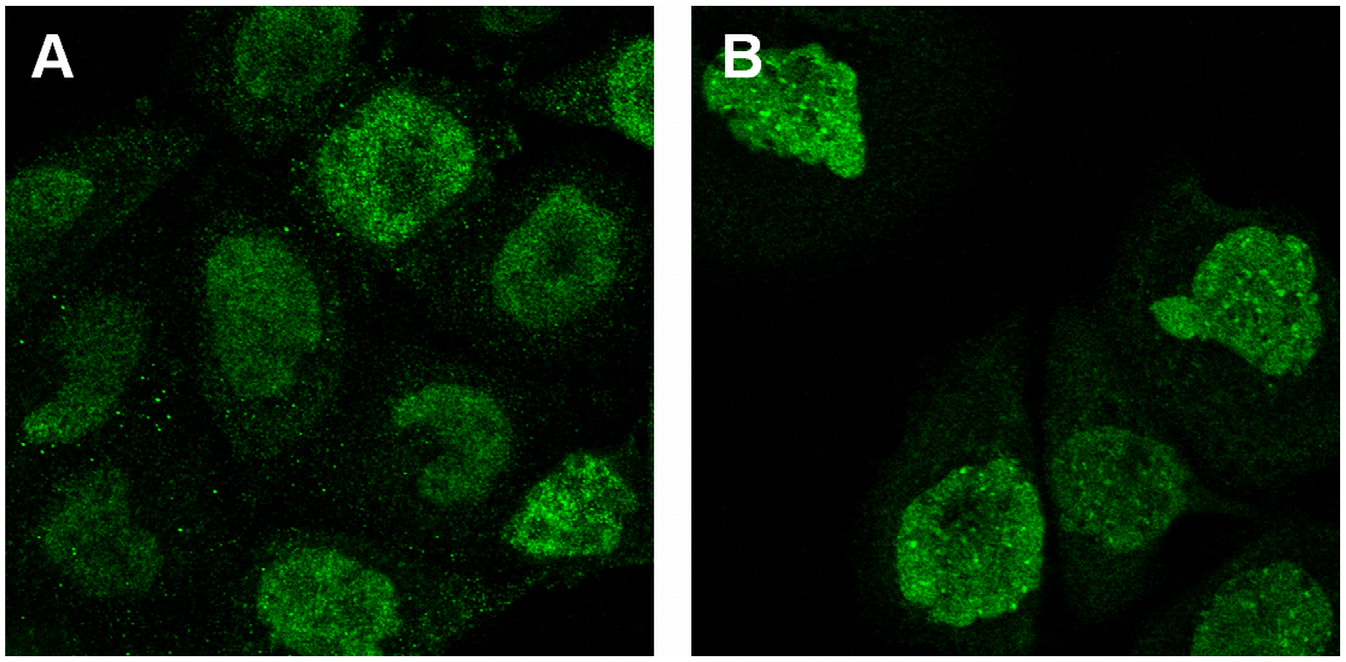
Examples of mis-annotated proteins identified by the hierarchical clustering reannotation method. (a) Protein “S100 calcium binding protein A12” was identified in the first round analysis. The image of the protein was visually annotated as “nucleus” but was annotated as “nucleus without nucleoli” by clustering. (b) Protein “Rho/Rac guanine nucleotide exchange factor (GEF) 2″ was identified in the second round analysis. The image of the protein was visually annotated as “nucleus” but was annotated as “nucleus without nucleoli” by clustering. In both cases the latter annotation was chosen upon re-examination.

We combined the lists of candidates obtained from the two methods above resulting in 106 (99+12–5 duplicates) proteins and provided them to the HPA team responsible for initial annotation of confocal images from the project. To enable estimation of the rate of annotation errors, we also included in the list 65 single class proteins obtained from random sampling (see Materials and Methods). Only the HPA index number was provided, so that the annotation team could not be influenced by the results from either the prior visual analysis or the automated analysis. After annotation of the total of 149 (106+65–22 duplicates) proteins, the labels were compared with those from the initial annotation and from classification or clustering. The results are summarized in **Table 2**. Of the proteins selected for reannotation by either classification or clustering, 41 proteins had their labels changed (the sum of the counts in the first and third columns for the first, second, fifth rows, and sixth row, minus 2 proteins present on both lists). These reannotated proteins are listed in **Table S1**. An image of a top ranked example from the proteins identified by SVM classification is shown in **Figure 2** **(a)**. **Figure 3** **(a)** shows the image of a top ranked example identified by clustering. These illustrate cases in which the automated approach resulted in correction of prior annotations.

**Table 2:** Summary of first round reannotation results.

	svm reannotation	random svm	clt reannotation	randomclt
AM right	21	4	2	4
partially right	7	5	0	1
both right	0	17	0	33
AM wrong	60	34	8	18
both wrong	9	4	1	8
Negative	2	1	1	1
Total	99	65	12	65

The column ‘svm reannotation’ includes the proteins identified by SVM classification reannotation method; the column ‘random svm’ includes the proteins randomly drawn; the column ‘clt reannotation’ includes the proteins identified by hierarchical clustering for reannotation; the column ‘random clt’ includes the proteins randomly drawn. The row ‘AM right’ indicates the proteins whose automated classified or clustered annotations were right, while the previous human annotations were wrong; the row ‘partially right’ indicates the proteins whose automated annotations were partially right, in that reannotation added the predicted annotation to the previous one; the row ‘both right’ indicates the proteins whose automated annotations were the same as previous human annotations, and where reannotation did not change it; the row ‘AM wrong’ indicates the proteins whose automated annotations were wrong, while the previous human annotations were right; the row ‘both wrong’ indicates the proteins whose automated annotations and previous human annotations were wrong, and a new assignment was made during reannotation. ‘Negative’ indicates those proteins that were reannotated as ‘non-specific location’ and designated for removal from the next release of the dataset. They could correspond to bad antibodies.

**Figure 4:**
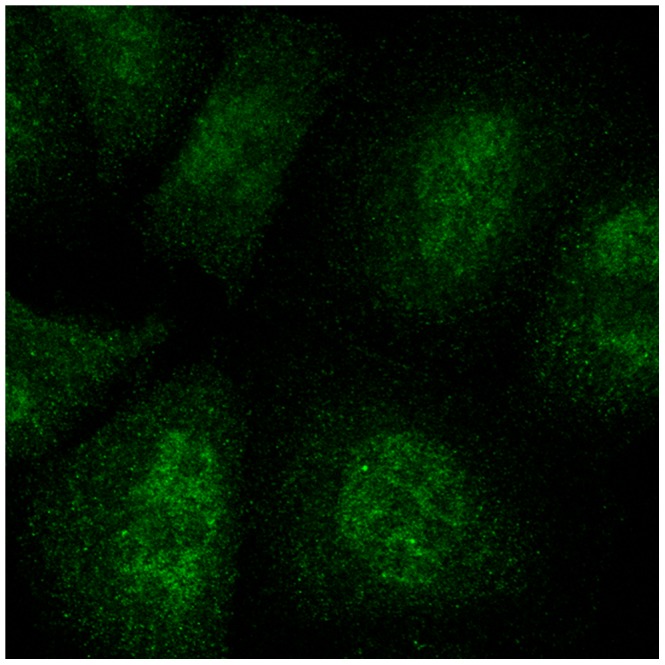
Example of detection of mixed patterns by clustering. Protein “nerve growth factor receptor” was visually annotated as “cytoplasm”, but was annotated as “nucleus without nucleoli” mixed with “cytoplasm” by clustering in the first round. The latter annotation was chosen after re-examination.

**Figure 5:**
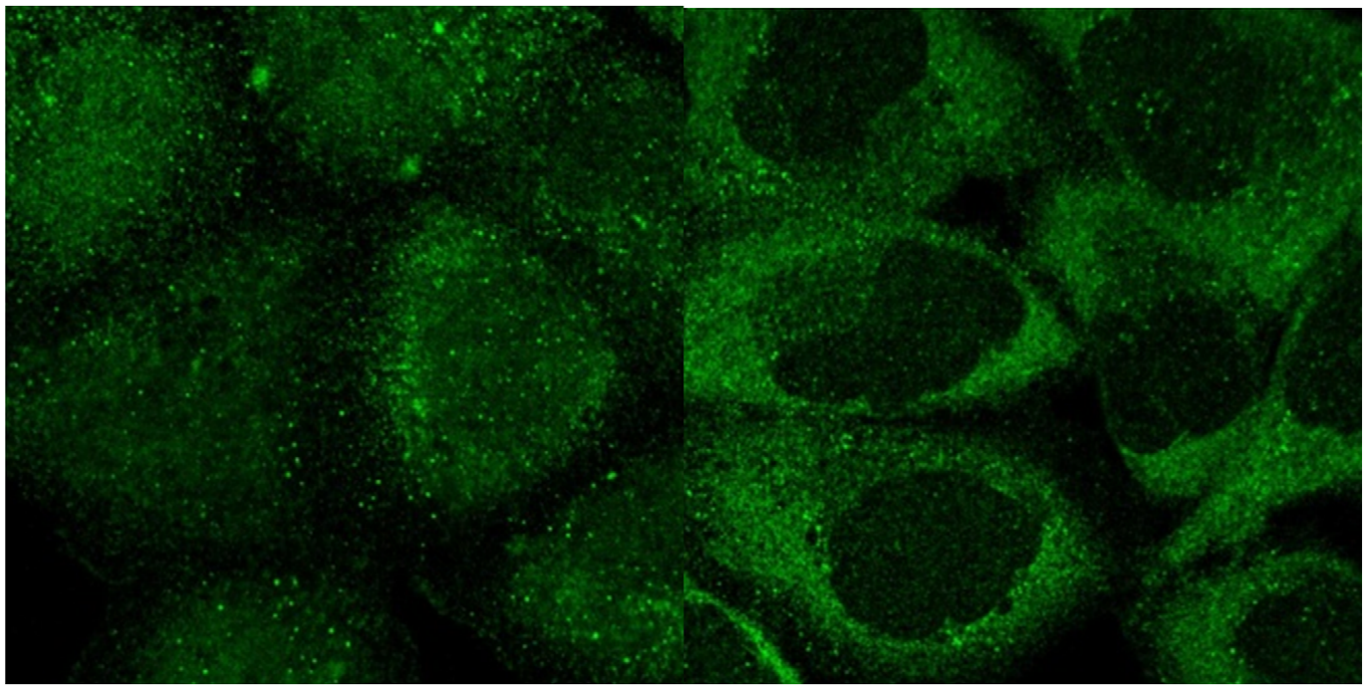
Example of sub-patterns identified by clustering. Proteins “neuronal pentraxin receptor” and “eukaryotic translation initiation factor 5″ were visually annotated as “cytoplasm,” but hierarchical clustering assigned them to separate clusters in the first round. The images indicate that they indeed display two cytoplasmic sub-patterns.

In addition to the possibility of single class proteins being incorrectly annotated, it was also possible that a protein showing more than one pattern might be incorrectly annotated as showing only a single pattern. An example of such a protein (identified by clustering) is shown in **Figure 4**. Furthermore, we can identify proteins annotated to the same location but that are clustered into different but nearly pure clusters, suggesting that they represent sub-patterns (**Figure 5**).

Given the results of Table 2, it was of interest to evaluate the yield of the two methods for finding proteins needing reannotation compared to that expected for random choice. Those entries in the first, second, fifth, and sixth rows of the table represent proteins whose annotations changed upon reexamination. The reannotation rate for proteins chosen at random was therefore 14/65 = 22%, while the rates for proteins identified by SVM and hierarchical clustering respectively were (21+7+11)/99 = 39% and (2+2)/12 = 33% (the rate for the combination of the two was 41/106 = 39%). Thus, we observed between 1.5-fold and 1.8-fold enrichment in identifying incorrectly annotated proteins above random.

Using the new annotations, we repeated the SVM classification. The resulting confusion matrix is shown in **Table S2**. The overall accuracy improved to 86.4% compared with 82.4% in Table 1. This improvement is due directly to the changes in the annotations of the re-examined proteins, and no improvement in classification of other proteins is observed.

### Second Round Reannotation

After incorporating the results from the first round analysis (i.e. some proteins reannotated), the same framework was applied on a new release (5.0) of HPA for a second round of analysis. The new dataset for the A-431 cell line contained images for 2749 proteins (extended and updated from release 4.0 used in the first round analysis), of which 958 were localized to one of thirteen major subcellular location patterns (classes): centrosome, cytoplasm, endoplasmic reticulum, Golgi, mitochondria, nucleoli, nucleus, nucleus without nucleoli, plasma membrane, vesicles, actin filaments, intermediate filaments and microtubules (the last three had previously been grouped under cytoskeleton). The number of proteins per class ranged from ten to 255. Most images of single class proteins in the first round were kept in the second round. However, a number of new proteins were added, some were removed, some proteins were reimaged and some proteins were reannotated based on the results of the first round. Given the updated dataset, we then repeated the approach used in the first round analysis for this second round. When SVM classifiers were trained and tested as before, we obtained the confusion matrix in **Table 3**, with an overall accuracy of 77.9%. Using the SVM classification method, a list of 156 proteins was generated as potentially mis-annotated. To reduce the burden of annotation work and make the whole process efficient, we selected a sub-list of 58 proteins of these using uniformly random selection. On the other side, we hierarchically clustered single pattern proteins into 119 clusters, with an accuracy of 66% when comparing their annotations with the dominant one of their cluster. Using a slightly different criterion (see Materials and Methods), 63 proteins were identified for re-examination. We combined the two lists into one with 103 (58+63–18 duplicates) proteins and merged it with 80 proteins from random sampling (see Materials and Methods). As a result, in total 162 (103+80–21 duplicates) proteins were again subjected to reannotation. The validation results and statistics are presented in **Table 4**. 31 proteins were reannotated (see **Table S3**). Two images of top ranked representative examples from the proteins generated both by SVM classification for reannotation and by clustering for reannotation are shown in **Figure 2** **(b)** and **Figure 3** **(b)** respectively.

**Table 3:** Classification results before second round of reannotation.

	centro.	cyto.	actin	inter.	micro.	er	golgi	mitoch.	nucleoli	nucleus	w/o	PM	vesicle
Centrosome (16)	**0.38**	0.06	0	0	0	0	0.19	0	0	0.13	0	0	0.25
Cytoplasm (129)	0	**0.9**	0	0	0.01	0	0.01	0.03	0	0	0	0	0.05
Actin filaments (10)	0	0.6	**0**	0.1	0	0	0	0.1	0	0	0	0.1	0.1
Intermediate filaments (9)	0	0.33	0	**0.33**	0.11	0	0	0.22	0	0	0	0	0
Microtubules (21)	0	0.29	0	0	**0.67**	0.05	0	0	0	0	0	0	0
ER (41)	0	0.2	0	0	0	**0.68**	0	0.1	0	0.02	0	0	0
Golgi (64)	0	0.02	0	0	0	0	**0.86**	0.08	0	0	0	0	0.05
Mitochondria (148)	0	0	0	0.01	0	0	0.01	**0.96**	0	0	0	0	0.02
Nucleoli (67)	0	0	0	0	0	0	0	0	**0.87**	0.06	0.04	0	0.03
Nucleus (110)	0	0.01	0	0	0	0	0	0	0.05	**0.36**	0.58	0	0
Nucleus w/o nucleoli (255)	0	0	0	0	0	0	0	0	0.02	0.11	**0.87**	0	0
Plasma membrane (17)	0	0.59	0	0	0	0	0	0.06	0	0.06	0	**0.18**	0.12
Vesicles (71)	0.01	0.04	0	0	0	0.01	0.04	0.04	0	0.01	0	0	**0.83**

Cell level feature classification confusion matrix. Bold values indicate agreement between the classifier and the true class. Overall classification accuracy is 77.9%. The number of proteins in each class is shown in parenthesis after the class name.

**Table 4:** Summary of second round reannotation results.

	svm reannotation	random svm	clt reannotation	randomclt
AM right	14	2	14	2
partially right	3	6	0	3
both right	0	48	0	29
AM wrong	37	23	43	42
both wrong	2	1	5	4
Negative	2	0	1	0
Total	58	80	63	80

See legend to Table 2 for definitions of row and column headings.

The results of Table 4 indicate that the reannotation rate for proteins chosen at random was 9/80 = 11% and the rates for SVM and hierarchical clustering respectively were (14+3+2+2)/58 = 36% and (14+5+1)/63 = 32%. Hence the enrichment of automated methods was between 2.9-fold and 3.3-fold above random. The subset of proteins chosen for reannotation by *both* methods showed an enrichment 5-fold above random (10/18 = 55%). Upon retraining the SVM classifier with the reannotations and the resulting 950 single pattern proteins, the overall accuracy increased to 82.3% (**Table 5**). Unlike the first round, this improvement is attributed both to the changes in the annotations of the re-examined proteins, and to correctly classifying a few additional proteins with the improved classifier.

**Table 5:** Classification results after second round of reannotation.

	centro.	cyto.	actin	inter.	micro.	er	golgi	mitoch.	nucleoli	nucleus	w/o	PM	vesicle
Centrosome (16)	**0.31**	0.06	0	0	0	0	0.19	0.13	0.06	0.06	0	0	0.19
Cytoplasm (126)	0	**0.94**	0	0	0	0	0	0.02	0	0	0	0	0.04
Actin filaments (10)	0	0.40	**0.10**	0.10	0	0	0	0.10	0	0	0	0.20	0.10
Intermediate filaments (12)	0	0.25	0	**0.42**	0	0.08	0	0.25	0	0	0	0	0
Microtubules (18)	0	0.17	0	0	**0.78**	0.06	0	0	0	0	0	0	0
ER (40)	0	0.13	0	0	0	**0.78**	0	0.10	0	0	0	0	0
Golgi (64)	0	0.02	0	0	0	0	**0.97**	0	0	0	0	0	0.02
Mitochondria (148)	0	0.01	0	0.01	0	0.01	0.01	**0.95**	0	0	0.01	0	0.01
Nucleoli (66)	0	0.02	0	0	0	0	0	0	**0.88**	0.05	0.03	0	0.03
Nucleus (91)	0	0	0	0	0	0	0	0	0.07	**0.30**	0.64	0	0
Nucleus w/o nucleoli (272)	0	0	0	0	0	0	0	0	0.01	0.04	**0.94**	0	0
Plasma membrane (14)	0	0.50	0	0	0	0	0	0.07	0	0.07	0	**0.29**	0.07
Vesicles (73)	0	0.05	0	0	0	0	0.01	0.07	0	0.03	0.01	0	**0.82**

Cell level feature classification confusion matrix with reannotated proteins. Bold values indicate agreement between the classifier and the true class. Overall classification accuracy is increased to 82.3% compared with 77.9% in Table III. The number of proteins in each class is shown in parenthesis after the class name.

### Identifying Single Pattern Proteins in Mixed Collections

The frequency of changes in annotations observed when re-examining randomly selected proteins in the two rounds (11–22%) indicates that the reproducibility, and likely the accuracy, of such assignments is approximately 89–94% which was calculated from 1– (22%)/2 or 1– (11%)/2 (assuming that the probability of error on reannotation is independent of whether an error had been made originally). Given that the accuracy estimated for the SVM classifier (77.9–86.4%) is similar to this when considering just single class proteins (as we can do when using it for reannotation since initial labels are available), we sought to determine whether a similar accuracy could be obtained when considering all proteins (as would be required if doing initial annotations). We considered two variations on this test. We used a dataset consisting of 2749 proteins (after some reannotations in the second round) in 77 classes of single and mixed patterns that contain at least 5 proteins (a total of 46739 cells).

**Figure 6:**
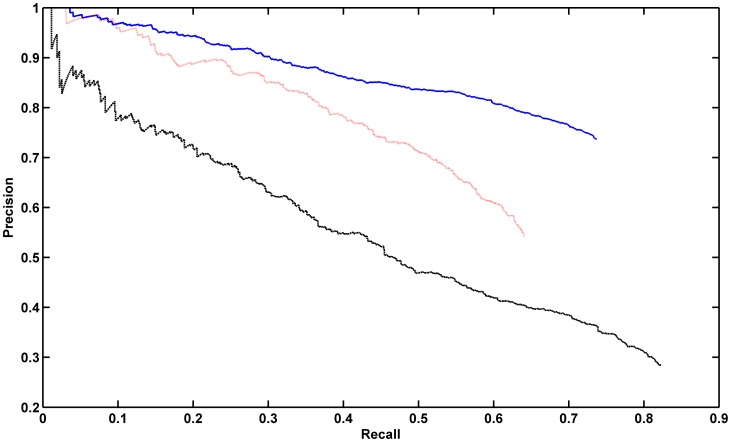
Precision-recall curves for protein annotations for single and multi-class classifiers. For the solid blue and dashed black line, we predicted the annotations of single pattern and mixed pattern proteins used a classifier trained with only single pattern proteins. For the solid blue line, annotations were considered correct if one of the annotations of one protein was predicted. For the dashed black line, only recognition of single pattern proteins was considered correct. For the dotted red line, a classifier trained on both single and mixed pattern proteins was used, but only the accuracy of recognizing single pattern proteins was assessed.

In the first variation, we applied the single pattern classifier to all proteins (including mixed pattern proteins) and determined how accurately it could assign at least one correct label and how accurately it could recognize proteins with just a single class. We split the 950 single pattern proteins into 5 folds (ensuring that all images for a given protein were in the same fold) and also split the mixed pattern proteins into 5 folds. Every four single pattern folds were used to train a classifier (the training set was further divided for tuning parameters as described in the Materials and Methods), and it was used to assign labels to the remaining fold of single pattern proteins and one of the multiple pattern folds. After classification we performed precision-recall analysis, which determines accuracy of the classifier as a function of the confidence that it estimates for each prediction. We assessed how we would recognize at least one of the labels for multiple-class proteins. This is demonstrated by the solid blue curve in **Figure 6**. The precision was defined in this case as the number of proteins correctly or partially correctly classified with probability above a varying threshold divided by the number of proteins classified with probability above that threshold, and the recall as the number of proteins correctly or partially correctly classified with probability above the threshold divided by total number of proteins. With a zero threshold, we could correctly assign at least one annotation for 73.7% of the proteins. As the threshold is increased (reducing the recall), the accuracy rises almost linearly. We then considered the effect of requiring that all labels be correctly assigned. In this case, all multiple-class proteins are by definition incorrectly classified, and we seek to determine whether single class proteins can be recognized by the single-class classifier with higher confidence than multiple-class proteins. As shown in **Figure 6** (dashed black line), the system with zero threshold obtained an overall precision of 28.5%. The precision in this case was defined as the number of single pattern proteins correctly classified with probability above the threshold divided by the number of all proteins classified with probability above the threshold, and recall was defined as the number of single pattern proteins correctly classified with probability above threshold divided by the total number of single pattern proteins. When the threshold was increased to obtain a recall of 60%, the classification accuracies increased to only 42.0%. Thus, we cannot use the single pattern classifier to find single-class proteins in a set of proteins with no annotations (e.g., a new batch of images). However, the previous results show that we can still assign one label to both single-class and multiple-class proteins with good precision.

In a second variation, we retrained the SVM classification framework with classes consisting of all label combinations observed for both single and mixed patterns (there were 77 unique classes), explicitly giving it the ability to recognize single class proteins in the presence of multiple-class proteins. The overall accuracy of the classifier for all patterns was only 45.4%, illustrating the difficulty of assigning all labels correctly. We therefore asked how well the single protein classes could be recognized. The precision-recall curve for this task is shown as the dotted red line in **Figure 6**. The precision was defined as the number of single pattern proteins correctly classified with probability above the threshold divided by the number of proteins classified as single pattern with probability above the threshold, and recall as the number of single pattern proteins correctly classified with probability above the threshold divided by the total number of single pattern proteins. At a zero threshold, the accuracy for recognizing single class proteins was found to be 64.0%. At a threshold corresponding to 17% recall, the precision improved to 90.1%. Thus single class proteins can be correctly recognized with reasonable accuracy by a classifier trained on either single or multiple-class proteins.

## Discussion

Microscopy images are rich sources of information about cell structure and function for systems biology. We have presented a framework to classify proteome-scale collections of proteins containing complex subcellular location patterns, and our classifier provides performance similar to human annotation on single-class proteins.

The only prior work on the automated classification of proteins using HPA confocal immunofluorescence images was described by Newberg et al. [5]. In this paper, we obtain similar classification accuracies on single-class proteins but analyze many more proteins and patterns. The cytoplasm pattern, which has the second largest number of proteins, was added and introduces some confusion with other patterns because of non-specific staining over the cell. The nucleus pattern was split into nucleus pattern and nucleus without nucleoli pattern to provide more detailed annotations, notwithstanding the two are highly blended in the staining and are difficult to distinguish visually in many images. The small class of cytoskeleton was also even split into three further patterns of actin, intermediate filaments and microtubules which reduces the number of training images available for each. Nonetheless, good classification accuracies were maintained, which represents a significant advance over our prior work. However, the accuracies are not yet high enough to replace human annotators. In the future, we plan to implement new features specific for the centrosome pattern, and hope to add features for better discriminating the cytoskeleton and plasma membrane patterns from the cytoplasm pattern.

One of the main novelties we describe in this paper is the introduction of approaches to identify possible mis-annotated proteins, derived from SVM classification and hierarchical clustering, and the demonstration that they could identify proteins needing reannotation at a rate higher than random. Our results show that selecting proteins using *both* schemes achieves higher yield of reannotated proteins than either of them alone or in combination. We plan to continue cycles of reannotation, and to incorporate the automated system in the annotation pipeline. Note that in this paper we only provide results for the A-431 cell line, but the whole framework introduced here can be applied to other cell lines, such as U-2OS and U-251MG. As a matter of fact, some preliminary results have already been obtained (data not shown; included in Reproducible Research Archive as described in Materials and Methods). We hope thereby to maximize the accuracy of reported annotations in the Human Protein Atlas. We anticipate that a similar approach may be applied to other proteome-scale image collections.

The dataset used in this paper contains 2D, static confocal images of fixed cells from HPA. In the future, the temporal dynamics of the variations of protein subcellular location patterns and the evolution over the course of stem cell differentiation can be explored by our framework as datasets become available.

Another novel aspect of this work is the results on full or partial recognition of mixed pattern proteins. Our results highlight the difficulty of handling these patterns. The main problem is that the features are affected by the degree of mixture. This is unlike the case for tasks like document classification, in which the addition of a second topic associated with new words does not alter the detection of words associated with the first topic. It is also unlike the case in many natural scene images in which adding a dog to an image of a cat does not change the local features associated with the cat. In these cases, a number of multiclass learning strategies have been successfully used. For protein patterns consisting of vesicular objects, we have used similar methods to show that the frequency of object types can be used to estimate mixing between patterns (using both supervised [13] and unsupervised [14] approaches). Unfortunately, this approach does not generalize to mixtures involving non-vesicular proteins, and preliminary work indicates that local features such as SIFT [15] also do not perform well in that case.

## Materials and Methods

### Image Collection

Confocal images of A-431 cells from the HPA were used for these studies. These images are stored as one 8-bit uncompressed TIFF file for each of four fluorescence channels. One channel was collected for immunofluorescence labeling with monospecific antibodies, while the other channels were acquired using standard stains for the nucleus, endoplasmic reticulum and microtubule cytoskeleton [11]. After images were acquired, they were visually annotated. One or more location labels were assigned to each protein (i.e., a protein could be viewed as consisting of a centrosome pattern mixed with cytoplasm pattern). Up to two image fields were taken for each protein.

### Cell Segmentation and Feature Calculation

We used the same cell segmentation and feature calculation strategies as in our previous work [5]. The result was a total of 714 features for each cell, for an average of 9 cells per image. The much larger number of features compared to cells in each class suggested the need for some feature reduction or selection method, and we chose Stepwise Discriminant Analysis as it has worked well in this field of application [16]. After selection there were around 100 features left.

### Support Vector Machine Classification

We trained SVM to classify cells by their subcellular location patterns in two rounds. We utilized two levels of nested 5-fold cross-validation so that training parameters could be optimized without using the final testing data. The fraction of representatives of each class within each fold was kept as close to the original fractions as possible, and all cells for a given protein were included in the same fold to give the most conservative estimate of classification performance. The inner level of cross validation involved using 3 folds for training and one fold for selecting the optimal values of the radial basis function (RBF) kernel parameter g and the slack parameter C; the outer level used the remaining fold to get the final generalization accuracies. Additionally, class weights were used during training in order to account for the different number of cells in representing each class. Classification was implemented using the LIBSVM toolbox [17] (http://www.csie.ntu.edu.tw/~cjlin/libsvm) with one-against-one multi-class SVM (unless otherwise indicated). Since the classifiers output probabilities that each cell belongs to the classes, we boosted the classification accuracy of single cells by summing class probabilities for all cells for the same protein, and then assigning all of these cells the class with the maximum value.

For identifying potential proteins that may need to be reannotated, we designed an algorithm on the basis of the output probabilities estimated by SVM classifiers. From the output probabilities, we find a set of samples (we call set R) that are incorrectly classified but have low predicted probabilites. These samples are near to the decision boundaries. On the other hand, there is another set of samples (set F) that are also incorrectly classified but with higher predicted probabilities, which are farther away from decision boundaries. The fundamental idea of the algorithm is that R has little impact on F. Even if we flip the labels of samples in R from their previous class labels to the classified labels and train the classifiers again, at least a subset of samples in F will still be stable and stay in the status of incorrectly classified. Therefore F are identified as potentially being incorrectly annotated. This algorithm is nonparametric and robust, and bears an analogy to the distillation process. The detailed procedure follows: (1) find the proteins whose automated and human classes disagree and sort them in ascending order of classifier-assigned probability; (2) change the annotations for the top N (we used N = 5) proteins in this ranked list to match the automated assignment (so that all combinations of changes of these labels are considered), and (3) retrain the classifiers and repeat steps 1 and 2 for M (we used M = 20) levels of recursion. At the end of this process, the proteins that appeared in all ranked lists were considered for reannotation.

In addition to using the classifier for reannotation, we sought to determine how well it could be used for initial annotation of proteins. In this case, we do not know a priori which proteins show single patterns and which show mixed ones. We applied the classifier (trained on only the single pattern proteins) to images for 2749 proteins after the second round of reannotation with single or mixed patterns which have at least 5 proteins, and sorted the proteins by the magnitude of the maximum output probability value for each protein. An increasing threshold on this probability was used to generate precision-recall curves using two approaches for defining precision and recall. In the first case of variation, we defined correct classifications as assigning at least one of a protein’s labels correctly with probability above the threshold. In the second case, we defined only assignments (with probability above the threshold) to *single class* proteins as correct (and thus all assignments above the threshold made to proteins with two or more labels were considered incorrect).

In our preliminary work on classification of subcellular location patterns using HPA images [5], a subset of images of single pattern proteins were evaluated by both SVM and Random Forest [18] methods. The results indicated slightly better performance for the latter approach, and we therefore also evaluated Random Forest classifiers for the tasks on the larger datasets used in this paper. Since the performance was lower than for SVM (as shown in **Table S4**), we used SVMs throughout this paper.

### Hierarchical Clustering for Reannotation

As an alternative to classification (which requires labels for training), we used an unsupervised machine learning method, hierarchical clustering, to identify candidate proteins for reannotation in two rounds. For this we used the same features and a normalized Euclidean distance metric with Stepwise Discriminant Analysis feature selection. Since there was more than one cell for each protein (and some of these might be atypical), we chose the cell closest to the multivariate median normalized feature value for a given protein to represent that protein in the clustering. The resulting tree can be cut at various values of the distance measure to give different numbers of clusters. We defined the cluster annotation for each protein as the dominant human annotation in the cluster in which the protein is found.

To choose the optimal number of clusters, Akaike information criterion was used. It balances the log-likelihood of the data given the clustering against the number of clusters. After we decided the clustering of proteins, the clusters were ordered by optimal leaf ordering [19] using the associated annotations.

Once we obtained the clustering of proteins, we computed two scores for each protein to measure and identify the proteins whose annotations might be not correct. The first score is the ratio of the number of proteins of that protein’s class in its cluster to the number of proteins in the dominant (plurality) class of that cluster; the smaller the ratio is, the higher confidence the protein is wrongly annotated. The second score is the normalized feature distance of each protein to the “median feature vector” of proteins in that protein’s cluster which have the dominant annotation; a small distance means that the protein is likely to be correctly clustered. In the first round, we found a subset of all proteins with the below one value of the first score (in total 285 lowest scores by the first definition) and another subset of proteins with the 300 lowest scores by the second definition (which were from the range between zero and the value around the peak of the histogram of the second score, data not shown), and then selected proteins in the intersection of the two subsets as candidates for reannotation. However, we restricted the final list by requiring that each cluster could only have one protein in this list to minimize the effect that the presence of more than one mis-annotated protein might have on the quality of a cluster. In the second round, we released these restrictions. Proteins were sorted with the first score and with the second score respectively in ascending order; then they were sorted with the sum of the two ranks ascendingly. As a result, we had all proteins sorted in one list, and the more confidence we had on one protein for its being incorrectly annotated, the higher it would be in the sorting. The final subset of proteins that would be reexamined by annotators was thus generated from the top until we thought that the number of proteins in the subset would not be an inappropriate burden of work for the annotators.

### Random Sampling for Reannotation

To serve as a baseline for evaluating the reannotation enrichments we would obtain from automated methods (SVM and hierarchical clustering), we created another list of proteins to be reexamined. Due to the highly imbalanced dataset, we made a compromise schema for the random sampling. For each class, we uniformly randomly sampled a small number (r) of proteins *with replacement*. Thus we were easily able to ensure that we sampled proteins from all classes especially those with small size and meanwhile to control the number of proteins in this list to reduce the burden of reannotation work. On the other hand, we could reduce the chances of selecting the majority (or even all) proteins from some small classes with replacement sampling. Then the unique set of proteins (without the duplicates) was merged with those identified from the automated methods and subjected to reexamination. In both rounds of analyses, we used r  = 7 proteins for each class for a reasonable and acceptable number of proteins.

### Reproducible Research Archive

All code and intermediate results for the work described here will be available as an open source reproducible research archive from http://murphylab.web.cmu.edu/software upon publication.

## Supporting Information

Table S1 **List of proteins reannotated after first round.**
(DOC)

Table S2 **Classification results after first round of reannotation.**
(DOC)

Table S3. **List of proteins reannotated after second round.**
(DOC)

Table S4 **Classification results using Random Forest classifier after second round of reannotation.**
(DOC)
