# Supplementary material for: Automated Analysis and Reannotation of Subcellular Locations in Confocal Images from the Human Protein Atlas
Source: PLoS One. 2012 Nov 30;7(11):e50514. doi: 10.1371/journal.pone.0050514 (PMC3511558; doi:10.1371/journal.pone.0050514)
Supplement: Table S1 — List of proteins reannotated after first round. (DOC) [file pone.0050514.s001.doc]

**Table S1 list of proteins reannotated after first round**

| **Protein Index in HPA** | **Protein Name** | **Previous human annotations** | **Automated annotations** | **Reannotated annotations** |
| --- | --- | --- | --- | --- |
| **230** | Plexin-B3 | Nucleus | Nucleus without nucleoli | Nucleus without nucleoli |
| **238** | Ankyrin repeat and SOCS box protein 11 | Nucleus | Nucleus without nucleoli | Nucleus without nucleoli |
| **325** | Armadillo repeat-containing X-linked protein 3 | Cytoskeleton | Cytoplasm | Cytoskeleton, centrosome |
| **390** | Autism susceptibility gene 2 protein | Nucleus | Nucleus without nucleoli | Nucleus without nucleoli |
| **447** | H/ACA ribonucleoprotein complex subunit 4 | Nucleus | Nucleoli**/**Golgi | Nucleoli |
| **604** | Uncharacterized protein C22orf31 | Nucleus | Nucleus without nucleoli | Nucleus without nucleoli |
| **634** | Immunoglobulin-binding protein 1 | Cytoskeleton | Cytoplasm | Cytoskeleton, centrosome |
| **846** | Uncharacterized protein KIAA0586 | Nucleus | Nucleus without nucleoli | Nucleus without nucleoli |
| **1242** | Retrotransposon gag domain-containing protein 1 | Nucleus | Nucleus without nucleoli | Nucleus without nucleoli |
| **1306** | DnaJ homolog subfamily A member 1 | Cytoskeleton | Cytoplasm | Cytoplasm, cytoskeleton |
| **1380** | Cadherin-13 | Plasma membrane | Cytoplasm | Negative |
| **1549** | Galectin-7 | Cytoskeleton | Cytoplasm | Negative |
| **1662** | Sex comb on midleg-like protein 2 | Cytoplasm | Nucleus without nucleoli**/**Cytoplasm | Cytoplasm, nucleus |
| **1894** | G patch domain and KOW motifs-containing protein | Nucleus | Nucleus without nucleoli | Nucleus without nucleoli |
| **1923** | Transcription factor SOX-6 | Nucleus | Nucleus without nucleoli | Nucleus without nucleoli |
| **2082** | von Willebrand factor | Nucleus | Nucleus without nucleoli | Nucleus, nucleoli |
| **2185** | Cullin-5 | Golgi | Nucleus without nucleoli**/**Cytoplasm | Golgi, nucleus without nucleoli |
| **2543** | Thioredoxin domain-containing protein 16 | Mitochondria | Cytoplasm**/**Mitochondria | Cytoplasm, mitochondria |
| **2640** | RAF proto-oncogene serine/threonine-protein kinase | Nucleus | Nucleoli | Nucleus without nucleoli |
| **2881** | Protein S100-A12 | Nucleus | Nucleus without nucleoli | Nucleus without nucleoli |
| **3044** | Thiosulfate sulfurtransferase | Cytoskeleton | Mitochondria | Mitochondria |
| **3099** | Tetratricopeptide repeat protein 28 | Mitochondria | Cytoplasm | Mitochondria, nucleoli |
| **3111** | Uncharacterized protein C14orf43 | Nucleus | Nucleus without nucleoli | Nucleus without nucleoli |
| **3310** | Tetratricopeptide repeat protein 8 | Nucleus | Nucleus without nucleoli | Nucleus without nucleoli |
| **3473** | G antigen family B member 1 | Cytoskeleton | Cytoplasm | Cytoplasm, nucleoli |
| **4689** | Rho GTPase-activating protein 1 | Lys/pero/endo | Cytoplasm | Lys/pero/endo, centrosome |
| **4765** | Tumor necrosis factor receptor superfamily member 16 | Cytoplasm | Nucleus without nucleoli | Cytoplasm, nucleus without nucleoli |
| **4899** | B-cell lymphoma 6 protein | Nucleus | Nucleus without nucleoli | Nucleus without nucleoli |
| **5688** | Caspase-8 | Mitochondria | Cytoplasm | Cytoplasm, centrosome, mitochondria |
| **5701** | PRELI domain-containing protein 1, mitochondrial | Mitochondria | Cytoplasm | Mitochondria, nucleus |
| **5747** | Protein AF-10 | Nucleus | Nucleus without nucleoli | Nucleus without nucleoli |
| **6314** | Poly(A)-specific ribonuclease PARN | Nucleus | Nucleus without nucleoli | Nucleus without nucleoli |
| **6672** | Zinc finger protein with KRAB and SCAN domains 1 | Nucleus | Nucleus without nucleoli | Nucleus without nucleoli |
| **6680** | Neuroligin-1 | Nucleus | Nucleus without nucleoli | Nucleus without nucleoli |
| **6973** | Uncharacterized | Nucleus | Nucleus without nucleoli | Nucleus without nucleoli |
| **7927** | Spectrin alpha chain, brain | Cytoskeleton | Cytoplasm | Cytoskeleton, lys/pero/endo |
| **8066** | Caldesmon | Cytoskeleton | Cytoplasm | Plasma membrane, cytoskeleton |
| **8333** | Delta-1-pyrroline-5-carboxylate synthetase | Mitochondria | Golgi | Lys/pero/endo |
| **9064** | Zinc finger protein 134 | Nucleus | Nucleus without nucleoli | Nucleus without nucleoli |
| **9067** | Serine protease inhibitor Kazal-type 5 | Golgi | Cytoplasm | Cytoplasm, golgi |
| **10698** | Sodium/potassium-transporting ATPase subunit beta-2 | Nucleus | Nucleus without nucleoli**/**Nucleus | Nucleus without nucleoli |

The ‘Automated annotations’ column shows annotations that were assigned by SVM classification and hierarchical clustering; if they did not agree, both are shown (in that order) separated by “/”.
