# Supplementary material for: Automated Analysis and Reannotation of Subcellular Locations in Confocal Images from the Human Protein Atlas
Source: PLoS One. 2012 Nov 30;7(11):e50514. doi: 10.1371/journal.pone.0050514 (PMC3511558; doi:10.1371/journal.pone.0050514)
Supplement: Table S2 — Classification results after first round of reannotation. (DOC) [file pone.0050514.s002.doc]

**Table S2 Classification results after first round of reannotation**

|  | *centro.* | *cyto.* | *cytosk.* | *Er* | *golgi* | *l/p/e* | *mitoch.* | *nucleoli* | *nucleus* | *nucw/onucleoli* | *PM* |
| --- | --- | --- | --- | --- | --- | --- | --- | --- | --- | --- | --- |
| Centrosome (12) | **0.58** | 0.08 | 0 | 0 | 0.17 | 0.08 | 0 | 0 | 0 | 0.08 | 0 |
| Cytoplasm (324) | 0 | **0.99** | 0 | 0 | 0 | 0 | 0 | 0 | 0 | 0 | 0 |
| Cytoskeleton (29) | 0 | 0.48 | **0.45** | 0 | 0 | 0 | 0.07 | 0 | 0 | 0 | 0 |
| Er (34) | 0 | 0.18 | 0.03 | **0.71** | 0 | 0 | 0.09 | 0 | 0 | 0 | 0 |
| Golgi (39) | 0 | 0.03 | 0 | 0 | **0.95** | 0 | 0.03 | 0 | 0 | 0 | 0 |
| lys/pero/endo (26) | 0.04 | 0.19 | 0 | 0 | 0.12 | **0.54** | 0.12 | 0 | 0 | 0 | 0 |
| Mitochondria (100) | 0 | 0.1 | 0 | 0 | 0.01 | 0 | **0.89** | 0 | 0 | 0 | 0 |
| Nucleoli (38) | 0 | 0 | 0 | 0 | 0 | 0 | 0 | **0.89** | 0.03 | 0.08 | 0 |
| Nucleus (65) | 0 | 0 | 0 | 0 | 0 | 0.02 | 0 | 0.03 | **0.32** | 0.63 | 0 |
| nucleus w/o nucleoli (187) | 0 | 0 | 0 | 0 | 0 | 0 | 0 | 0.01 | 0.02 | **0.97** | 0 |
| Plasma membrane (6) | 0 | 0.5 | 0 | 0 | 0 | 0 | 0.17 | 0 | 0 | 0 | **0.33** |

Cell level feature classification confusion matrix with reannotated proteins. Bold values indicate agreement between the classifier and the true class. Overall classification accuracy is increased to 86.4% compared with 82.4% in Table I. The number of proteins in each class is shown in parenthesis after the class name.
