# Supplementary material for: Automated Analysis and Reannotation of Subcellular Locations in Confocal Images from the Human Protein Atlas
Source: PLoS One. 2012 Nov 30;7(11):e50514. doi: 10.1371/journal.pone.0050514 (PMC3511558; doi:10.1371/journal.pone.0050514)
Supplement: Table S3. — List of proteins reannotated after second round. (DOC) [file pone.0050514.s003.doc]

**Table S3 list of proteins reannotated after second round**

| **HPA Index** | **Protein Name** | **Previous human annotations** | **Automated annotations** | **Reannotated annotations** |
| --- | --- | --- | --- | --- |
| **593** | Methyl-CpG-binding protein 2 | Nucleus | Nucleus without nucleoli | Nucleus without nucleoli |
| **2018** | Platelet glycoprotein 4 | Golgi | Vesicles | Vesicles |
| **2732** | Zinc finger protein 544 | Nucleus | Nucleus without nucleoli | Nucleus without nucleoli |
| **3585** | Protein FAM50A | Nucleus | Nucleus without nucleoli | Nucleus without nucleoli |
| **4818** | DNA replication licensing factor MCM6 | Nucleus | Nucleus without nucleoli | Nucleus without nucleoli |
| **4911** | Heterogeneous nuclear ribonucleoprotein D0 | Nucleus | Nucleus without nucleoli | Nucleus without nucleoli |
| **6316** | Glypican-3 | Microtubules | Microtubules**/**Cytoplasm | Intermediate filaments |
| **6669** | Bcl-2-associated transcription factor 1 | Nucleus | Nucleus without nucleoli | Nucleus, nucleoli |
| **6749** | Ubiquitin carboxyl-terminal hydrolase 10 | Microtubules | Cytoplasm | Intermediate filaments |
| **7484** | Interleukin enhancer-binding factor 2 | Nucleus | Nucleus without nucleoli | Nucleus without nucleoli |
| **7845** | Calsequestrin-1 | Microtubules | Microtubules**/**Cytoplasm | Intermediate filaments |
| **8255** | Long-chain fatty acid transport protein 1 | Cytoplasm | Mitochondria**/**Vesicles | Negative |
| **8926** | Protein HEXIM1 | Nucleus | Nucleus without nucleoli | Nucleus without nucleoli |
| **11126** | Butyrophilin subfamily 1 member A1 | Cytoplasm | Vesicles | Vesicles |
| **12672** | Dachshund homolog 1 | Nucleus | Nucleus without nucleoli | Nucleus without nucleoli |
| **13331** | Myosin regulatory light chain 2, atrial isoform | Nucleus | Nucleus without nucleoli | Nucleus without nucleoli |
| **13775** | G-protein coupled receptor 15 | Nucleus | Nucleus without nucleoli | Nucleus without nucleoli |
| **13801** | CKLF-like MARVEL transmembrane domain containing 1 | Nucleus | Nucleus without nucleoli | Nucleus without nucleoli |
| **13903** | T-lymphoma invasion and metastasis-inducing protein 2 | Nucleus | Nucleus without nucleoli | Nucleus without nucleoli |
| **14347** | Probable mast cell antigen 32 homolog | Nucleoli | Nucleus**/** Nucleus without nucleoli | Negative |
| **14447** | Proline-rich transmembrane protein 2 | Cytoplasm | Golgi**/**Vesicles | Golgi |
| **14791** | Cleft lip and palate transmembrane protein 1-like protein | ER | Cytoplasm | ER, vesicles |
| **15327** | G protein-coupled receptor kinase 6 | Nucleus | Nucleus without nucleoli | Nucleus without nucleoli |
| **15801** | Probable histone-lysine N-methyltransferase NSD2 | Nucleus | Nucleus without nucleoli | Nucleus without nucleoli |
| **16438** | Zinc finger protein 185 | Plasma membrane | Cytoplasm | Plasma membrane, cytoplasm |
| **16930** | Pannexin-1 | Plasma membrane | Cytoplasm**/**Plasma membrane | Plasma membrane, cell junctions |
| **17046** | Rho/Rac guanine nucleotide exchange factor (GEF) 2 | Nucleus | Nucleus without nucleoli | Nucleus without nucleoli |
| **17653** | DnaJ homolog subfamily C member 14 | Nucleus | Nucleus without nucleoli | Nucleus without nucleoli |
| **18332** | Iroquois-class homeodomain protein IRX-6 | Plasma membrane | Vesicles**/**Plasma membrane | Plasma membrane, golgi, vesicle |
| **20027** | Ras association (RalGDS/AF-6) and pleckstrin homology domains 1 | Nucleus | Cytoplasm**/**Plasma membrane | Nucleus, cytoplasm |
| **21577** | Uncharacterized protein C9orf139 | Nucleus | Nucleus without nucleoli | Nucleus without nucleoli |

The ‘Automated annotations’ shows labels from SVM classification/hierarchical clustering; if they did not agree, both are shown.
