# Supplementary material for: Automated Analysis and Reannotation of Subcellular Locations in Confocal Images from the Human Protein Atlas
Source: PLoS One. 2012 Nov 30;7(11):e50514. doi: 10.1371/journal.pone.0050514 (PMC3511558; doi:10.1371/journal.pone.0050514)
Supplement: Table S4 — Classification results using Random Forest classifier after second round of reannotation. (DOC) [file pone.0050514.s004.doc]

**Table S4 Classification results using Random Forest classifier** after second round of reannotation

|  | *centro.* | *cyto.* | *actin* | *inter.* | *micro.* | *er* | *golgi* | *mitoch.* | *nucleoli* | *nucleus* | *w/o* | *PM* | *vesicle* |
| --- | --- | --- | --- | --- | --- | --- | --- | --- | --- | --- | --- | --- | --- |
| Centrosome (16) | **0.06** | 0.06 | 0 | 0 | 0 | 0 | 0.5 | 0.06 | 0 | 0.13 | 0 | 0 | 0.19 |
| Cytoplasm (126) | 0 | **0.9** | 0 | 0 | 0 | 0 | 0 | 0.02 | 0 | 0 | 0.01 | 0 | 0.07 |
| Actin filaments (10) | 0 | 0.4 | **0** | 0 | 0 | 0 | 0 | 0.3 | 0 | 0 | 0 | 0.1 | 0.2 |
| Intermediate filaments (12) | 0 | 0.42 | 0 | **0.08** | 0 | 0.17 | 0 | 0.33 | 0 | 0 | 0 | 0 | 0 |
| Microtubules (18) | 0 | 0.44 | 0 | 0 | **0.56** | 0 | 0 | 0 | 0 | 0 | 0 | 0 | 0 |
| ER (40) | 0 | 0.18 | 0 | 0 | 0 | **0.73** | 0 | 0.1 | 0 | 0 | 0 | 0 | 0 |
| Golgi (64) | 0 | 0.02 | 0 | 0 | 0 | 0 | **0.75** | 0.14 | 0.03 | 0 | 0 | 0 | 0.06 |
| Mitochondria (148) | 0 | 0.01 | 0 | 0 | 0 | 0.01 | 0 | **0.98** | 0 | 0 | 0 | 0 | 0.01 |
| Nucleoli (66) | 0 | 0 | 0 | 0 | 0 | 0 | 0 | 0 | **0.85** | 0.06 | 0.05 | 0 | 0.05 |
| Nucleus (91) | 0 | 0 | 0 | 0 | 0 | 0 | 0 | 0 | 0.1 | **0.22** | 0.67 | 0 | 0.01 |
| Nucleus w/o nucleoli (272) | 0 | 0 | 0 | 0 | 0 | 0 | 0 | 0 | 0.01 | 0.01 | **0.97** | 0 | 0 |
| Plasma membrane (14) | 0 | 0.5 | 0 | 0 | 0 | 0 | 0 | 0.07 | 0 | 0 | 0.07 | **0.07** | 0.29 |
| Vesicles (73) | 0 | 0.07 | 0 | 0 | 0 | 0 | 0.03 | 0.11 | 0 | 0.01 | 0.03 | 0 | **0.75** |

Cell level feature classification confusion matrix with reannotated proteins by Random Forest. Bold values indicate agreement between the classifier and the true class. Overall classification accuracy is 78.3% which is smaller than 82.3% as in Table V. The number of proteins in each class is shown in parenthesis after the class name.
